# Genetic Associations of Chronotype in the Finnish General Population

**DOI:** 10.1177/0748730420935328

**Published:** 2020-06-24

**Authors:** Mirkka Maukonen, Aki S. Havulinna, Satu Männistö, Noora Kanerva, Veikko Salomaa, Timo Partonen

**Affiliations:** * Department of Public Health Solutions, Finnish Institute for Health and Welfare, Helsinki, Finland; † Institute for Molecular Medicine Finland (FIMM/HiLIFE), Helsinki, Finland; ‡ Nightingale Health Oy, Helsinki, Finland

**Keywords:** chronotype, circadian rhythms, clock genes, genetic risk score, genome-wide association study, genotype, phenotype

## Abstract

Individuals with a later chronotype (evening types) tend to have unhealthier behaviors and increased morbidity and mortality as compared with those with an earlier chronotype (morning types). However, the role of genetics in explaining evening types’ adverse health and health behavior is unclear. Our aim was to study genetic associations of chronotype among 8433 Finns from the cross-sectional National FINRISK 2007 and 2012 studies. First, we studied associations between chronotype and 20 key clock genes with a candidate-gene approach and then performed a full genome-wide association study (GWAS) of chronotype. We also developed a genetic risk score (GRS) for chronotype based on 313 single nucleotide polymorphisms (SNPs) that have previously been associated with chronotype. Chronotype was assessed with a shortened version of Horne and Östberg’s Morningness-Eveningness Questionnaire (sMEQ), and for comparison, we also used the single self-evaluation question on chronotype from the questionnaire. Linear and logistic regression was used for statistical analysis assuming additive effects. The clock gene analysis revealed 1 independent association signal within *NR1D2* (lead SNP rs4131403) that was associated with chronotype (*p* < 0.05; as based on both chronotype assessment methods). The GWAS analysis did not yield any genome-wide significant associations (*p* > 5 × 10^−8^). However, higher GRS was associated with evening chronotype (*p* < 0.001; as based on both chronotype assessment methods). In conclusion, our findings indicated novel genetic associations between chronotype and the *NR1D2* clock gene, which has previously been associated with carbohydrate and lipid metabolism. Furthermore, the GRS was able to capture the genetic aspect of chronotype in our study population. These findings expand our knowledge of the genetic basis of chronotype.

The term *chronotype* has been generally applied to interindividual variation in the timing of daily rhythms, and because of this variation, individuals can be categorized from extreme morning (early) to extreme evening (late) types. In observational studies, chronotype can be assessed with validated questionnaires, such as the Morningness-Eveningness Questionnaire (MEQ), which yields a score based on individuals’ preferences for timing their daily activities (Horne and Östberg, 1976), and the Munich Chronotype Questionnaire (MCTQ), which yields a reference phase over the 24-h day based on the midpoint of sleep during work and free days (Roenneberg et al., 2007). Evening type has been associated with less healthy behaviors (e.g., smoking, lower physical activity) and dietary habits than morning types (Sato-Mito et al., 2011; Kanerva et al., 2012; Wennman et al., 2015; Patterson et al., 2016; Maukonen et al., 2017). Furthermore, evening types also bear a higher risk for morbidity (e.g., type 2 diabetes, cardiovascular diseases, and depressive disorder; Levandovski et al., 2011; Merikanto et al., 2013; Merikanto et al., 2015; Yu et al., 2015; Patterson et al., 2018; Knutson and von Schantz, 2018; de Punder et al., 2019) and premature all-cause mortality (Broms et al., 2014; Knutson and von Schantz, 2018; Didikoglu et al., 2019).

According to twin studies, genetic effects account for about 50% of the between-individual chronotype variance in adults (Koskenvuo et al., 2007; Barclay et al., 2010). In Finland, the estimate for additive genetic effects was 12% and for dominant genetic effects 38%, with the remainder accounted for by environmental factors not shared by siblings (Koskenvuo et al., 2007). The proportion of these influences is subject to a change across the life span in particular, between 36 and 64 years of age, as genetic effects on chronotype can become attenuated (Koskenvuo et al., 2007; Barclay et al., 2014). However, the role of genetics in explaining adverse health and health behavior associated with being an evening type is currently unclear.

Clock genes pose a strong candidate genes group for studying the genetic background of chronotype because they control the circadian rhythms and also play an important role in regulating energy homeostasis (Ribas-Latre and Eckel-Mahan, 2016; Reinke and Asher, 2019; Ashbrook et al., 2020). Consequently, a number of single nucleotide polymorphisms (SNP) of several clock genes (*CLOCK*, *CRY1*, *PER1*) have been associated with metabolic disturbances, obesity, and dietary habits (Ribas-Latre and Eckel-Mahan, 2016). However, previous candidate-gene approach studies on clock gene (e.g., *CLOCK*, *PER2*) associations of chronotype have been small-scale studies and yielded inconsistent results, as reviewed by von Schantz (2017). Furthermore, 4 genome-wide association studies (GWASs) of chronotype have identified a total of 351 independent loci associated with chronotype, including variants within clock genes, such as *CRY1*, *PER2*, and *PER3*, along with several other genes. These GWASs are based on 2 large cohorts, the 23andMe (Hu et al., 2016) and the UK biobank study (Jones et al., 2016; Lane et al., 2016), while the most recent one is a meta-analysis of these 2 cohorts (*n* = 697,828; Jones et al., 2019). These cohorts included a single self-evaluation question on chronotype. As for our data set, chronotype was assessed with 6 items of the original MEQ (Horne and Östberg, 1976) in addition to a single self-evaluation question, which increase the validity and specificity of the findings.

Our aim was to study the genetic associations of chronotype in the Finnish general population (*n* = 8433). First, we studied associations between chronotype and 20 known key clock genes, and we also attempted to replicate the previously reported associations within these genes and chronotype (e.g., Carpen et al., 2006; Etain et al., 2014; Song et al., 2016). Our second aim was to perform a full GWAS of chronotype. We also attempted to replicate the findings from the GWAS meta-analysis of chronotype (Jones et al., 2019). Our third aim was to develop a genetic risk score (GRS) for chronotype based on the 351 lead SNPs (SNPs with the smallest *p* values for certain locus) from the GWAS of Jones et al. (2019).

## Methods

### Study Population

This study included participants from the population-based cross-sectional National FINRISK 2007 (Vartiainen et al., 2010) and 2012 (Borodulin et al., 2015) studies. The FINRISK studies monitored trends in risk factors of noncommunicable diseases in the Finnish population and have been conducted every 5 years since 1972. Random samples (*n* = 9958 in 2007 and *n* = 9905 in 2012) of men and women were selected from the National Population Register covering the age groups between 25 and 74 years. The studies included self-administered questionnaires (e.g., questions on timing of daily activities and socioeconomic status) and a health examination (e.g., blood samples) conducted between January and March for FINRISK 2007 and between January and April for FINRISK 2012. Overall, 6258 persons participated in 2007 (participation rate 63%) and 5827 in 2012 (participation rate 59%). Our final sample included 8433 participants with chronotype and genetic data available.

FINRISK 2007 and FINRISK 2012 adhered to the guidelines of the Declaration of Helsinki. The Ethics Committee of the Hospital District of Helsinki and Uusimaa approved the research protocols. All participants signed the informed consent.

### Chronotype Assessment

We assessed chronotype using the following 2 methods: a shortened 6-item version of Horne and Östberg’s (1976) MEQ (sMEQ) and a single self-evaluation question on chronotype from the sMEQ.

#### sMEQ

The sMEQ included 6 items (items 4, 7, 9, 15, 17, and 19) from the original MEQ, accounting for 83% of the variance of the original (Hätönen et al., 2008; Suppl. Table S1). The answers to these items were scored according to the scoring of the original MEQ, and the final sum score of the sMEQ varied from 5 (extreme eveningness) to 27 (extreme morningness). In the analyses, the sum score was used either as a continuous (continuous sMEQ score) or as a binary variable (evening type: scores from 5 to 15, morning type: scores from 16 to 27; binary sMEQ score). In our study population, there were 7436 participants with complete information on genetics and on sMEQ, and they were included in the sMEQ analysis.

#### Single self-evaluation question on chronotype

In addition, we assessed chronotype with the single self-evaluation question on chronotype from the questionnaire (item 19): “There are so-called morning people and evening people, which are you?” (Suppl. Table S1). The question was used as a binary variable (single-item chronotype), in which those who replied either “rather more an evening than a morning type” or “definitely an evening type” were regarded as evening types, and those who replied “definitely a morning type” or “rather more a morning than an evening type” were regarded as morning types. In our study population, there were 8433 participants with complete information on genetics and on the single self-evaluation question, and they were included in the analysis of single-item chronotype.

In addition, we conducted a stepwise linear regression analysis to determine which of the 6 MEQ items (independent variables) explained the greatest proportion of variance in the final sum score of the sMEQ (dependent variable). The single item 19 (standardized β = −0.456) predicted 77% of the variance (adjusted *R*^2^ = 0.767) in the final sum score of the sMEQ. The single item 19 also correlated with the final sum score of the sMEQ (*r* = −0.876).

### Genotyping and Quality Control

At the FINRISK study sites, trained study nurses took whole-blood samples, which were stored at −70 °C. DNA was extracted at the Finnish Institute for Health and Welfare and genotyped in several batches at the Sanger Institute, Broad Institute, or Institute for Molecular Medicine Finland using the following Illumina GWAS arrays: HumanCoreExome, Omniexpress, and 610K. Our data included 5 batches, of which 4 were substudies of FINRISK 2007 (including COROGENE controls (610K), PREDICTCVD cases, and controls (Omniexpress); the rest were included in 2 batches with (HumanCoreExome) 1 batch including participants from FINRISK 2012 (HumanCoreExome). The same standard quality control methods and standard imputation procedures were centrally applied for the data from each platform, after which a joint quality control (minor allele frequency ≥0.05 [5%], Hardy-Weinberg equilibrium *p* > 1 × 10^−7^, imputed information score [INFO] >0.7, and missing proportion <0.02 [2%]) was performed to harmonize the data content. The genotyping and imputation were performed as described in Locke et al. (2019). For each cohort, before phasing, we performed batchwise genotype quality control using standard quality thresholds. We prephased the array genotypes with Eagle (v2.3; Loh et al., 2016) and imputed genotypes genome-wide with IMPUTE (v2.3.1; Howie et al., 2009) using 2690 sequenced Finnish genomes and 5092 sequenced Finnish exomes. We assessed imputation quality by confirming sex, comparing sample allele frequencies with reference population estimates, and examining imputation quality (INFO score) distributions. We excluded any variant with INFO <0.7 within a given batch from all replication/follow-up analyses. Furthermore, we also excluded closely related individuals (*n* = 125) from the final data set (PLINK pi_hat >0.20). In total, genotyping was performed for 5330 FINRISK 2007 participants and for 3439 FINRISK 2012 participants.

### Key Clock Genes

The clock gene analyses included 20 known key clock genes (*ARNTL*, *ARNTL2*, *BHLHE40*, *BHLHE41*, *CLOCK*, *CRY1*, *CRY2*, *CSNK1E*, *CSNK1D*, *NFIL3*, *NPAS2*, *NR1D1*, *NR1D2*, *PER1*, *PER2*, *PER3*, *RORA*, *RORB*, *RORC*, *TIMELESS*; Hayes et al., 2005; Takahashi, 2017; Sato et al., 2018; Kurien et al., 2019; Patke et al., 2019). Of the total 8668 SNPs within these clock genes, 4022 SNPs (Suppl. Table S2) passed the quality control and were included in the study. For the replication analyses, we selected altogether 66 SNPs that have previously been associated with chronotype within the following genes: *ARNTL*, *ARNTL2*, *CLOCK*, *CRY1*, *NFIL3*, *NPAS2*, *PER1*, *PER2*, *PER3*, *RORC*, and *TIMELESS* (Suppl. Table S3; (Katzenberg et al., 1998; Carpen et al., 2005; Mishima et al., 2005; Carpen et al., 2006; Matsuo et al., 2007; Lee et al., 2011; Kripke et al., 2014; Etain et al., 2014; Parsons et al., 2014; Song et al., 2016; Dmitrzak-Weglarz et al., 2016; Jankowski and Dmitrzak-Weglarz, 2017; Jones et al., 2019).

### Genome-wide Association Analyses

For the GWAS analyses, there were altogether 12,954,971 SNPs, of which 5,842,835 passed the quality control and were included in the GWAS analyses.

For the replication analysis, we selected the top 10,000 genome-wide associated SNPs reported in the GWAS of Jones et al. (2019), of which our data included 7741 after the quality control (Suppl. Table S4). The meta-analysis was based on 2 cohorts, the 23andMe and the UK biobank study, both of which included the self-evaluation question on chronotype. The 23andMe cohort included 2 identically worded questions: “Are you naturally a night person or a morning person?” The first of these identical questions included the following options: “night person,” “morning person,” “neither,” “it depends,” or “I’m not sure,” whereas the second of questions included the responses “night owl,” “early bird,” and “neither.” The UK Biobank used the following question: “There are so-called morning people and evening people, which are you?” with the following answer options: “definitely a ‘morning’ person,” “more a ‘morning’ than ‘evening’ person,” “more an ‘evening’ than a ‘morning’ person,” “definitely an ‘evening’ person,” “do not know,” and “prefer not to answer.” This question was a modification of the original MEQ item 19 (“One hears about ‘morning’ and ‘evening’ types of people. Which ONE of these types do you consider yourself to be?”).

### Genetic Risk Score

We developed a weighted GRS for chronotype based on 351 lead SNPs from the GWAS of Jones et al. (2019). By using external weights from an independent study, our model is less prone to be overfitted. Of the 351 SNPs, our data included 313 after the quality control (Suppl. Table S5). The GRS was created by summing the total number of minor alleles weighted by their corresponding regression coefficients for risk of being an evening type for our analysis based on the GWAS study (Jones et al., 2019) for each participant. For our analyses, we reversed the direction of the regression coefficients, since the coefficients were originally reported for risk of being a morning type. Furthermore, we also analyzed the individual associations of these 313 SNPs with chronotype.

### Statistical Analysis

All of the analyses have been conducted with linear (semicontinuous chronotype) and logistic (binary chronotype) regression models assuming additive effects and adjusted for age, sex, genotyping batch, and 5 first principal components to account for population stratification and other spurious effects. The *p* values were corrected for multiple testing with the Benjamini-Hochberg (BH) and Benjamini-Yekutieli (BY) false-discovery rate methods, with *p* values <0.05 considered significant in all analysis except for the full GWAS analysis of chronotype. For the full GWAS, analysis results were considered as genome-wide significant for *p* values <5 × 10^−8^ and suggestive for *p* values <1 × 10^−5^. Furthermore, in the clock gene analysis, significantly associated SNPs were further linkage disequilibrium (LD) clumped (using clump in PLINK, with a threshold *p* < 0.05, *r*^2^ > 0.5, range: 250 kb) based on BH- and BY-corrected *p* values to reveal independent association signals. With regard to GRS, we estimated the proportion of variance explained with adjusted partial *R*^2^ (continuous sMEQ) and partial pseudo *R*^2^ (Nagelkerke; binary chronotype), using the R package “rsq.”

Statistical analyses have been conducted with PLINK version 2.0 (Chang et al., 2015) and version 1.9 (LD clumping) and with the R statistical computing program, version 3.5.1 (R Core Team, 2018).

## Results

In our data, 32% of the participants were evening types (59% women) according to binary sMEQ score, whereas according to the single-item chronotype, 45% of the participants considered themselves as being more of an evening (52% women) than a morning type (Table 1). Furthermore, the evening types were on average 5 years younger than morning types in both chronotype assessments.

**Table 1.** Characteristics of the study population by chronotype with means and standard deviations (SD).

		Chronotype Measure			
		Binary sMEQ^a^ Score (*n* = 7436)		Single-Item Chronotype^b^ (*n* = 8433)	
	All	Morning	Evening	Morning	Evening
	*n* = 8433	*n* = 5072 (68%)	*n* = 2364 (32%)	*n* = 4674 (55%)	*n* = 3759 (45%)
sMEQ score, mean (SD)	17.6 (4.3)	20.0 (2.7)	12.6 (2.2)	20.6 (2.6)	13.9 (2.9)
sMEQ score, range	5-27	16-27	5-15	12-27	5-22
Age, mean (SD)	52.9 (14.0)	54.5 (13.2)	48.6 (14.6)	55.2 (13.1)	50.0 (14.6)
Female (%)	4404 (52%)	2602 (51%)	1384 (59%)	2446 (52%)	1958 (52%)

sMEQ = shortened Morningness-Eveningness questionnaire; ME score = Morningness-Eveningness score.

a. Chronotype based on the validated 6-item version of the MEQ.

b. Chronotype based on a single self-evaluation question of the MEQ.

### Key Clock Gene Associations of Chronotype

When *p* values were corrected for multiple testing with the BH method, altogether 124 SNPs within 3 clock genes (*CRY1*, *NFIL3*, *NR1D2* aka *Rev-erbβ*) were associated with continuous sMEQ score and single-item chronotype, whereas no associations were found by binary sMEQ score (Suppl. Table S2). These significant associations were further LD clumped (with threshold *p* < 0.05), which resulted in altogether 7 independent association signals. Within *CRY1*, 3 independent association signals with evening chronotype emerged. Two of the signals (lead SNPs rs8192440, with 39 correlated SNPs, and rs77706154) emerged with both continuous sMEQ score and single-item chronotype containing a correlated SNP (rs1017168A) that has previously been associated with evening type (Jones et al., 2019; Suppl. Table S3). One of the 3 signals within *CRY1* emerged solely with continuous sMEQ score (lead SNP rs3741891, with 43 correlated SNPs; Suppl. Table S2). Within *NFIL3*, 3 independent signals with morning chronotype emerged. One of the signals was found with both continuous sMEQ score (lead SNP rs2482702, with 5 correlated SNPs) and single-item chronotype (lead SNP rs9409419, with 6 correlated SNPs). One signal emerged solely with continuous sMEQ score (lead SNP rs2440590, with 4 correlated SNPs) and another one solely with single-item chronotype (lead SNP rs2440592, with 4 correlated SNPs) containing a correlated SNP (rs2482705A) that has previously been associated with morning type (Kripke et al., 2014; Suppl. Table S3). Within *NR1D2*, 1 independent association signal with evening chronotype emerged with both continuous sMEQ score and single-item chronotype (lead SNP rs4131403, with 21 correlated SNPs; Suppl. Table S2).

When the more conservative method of false-discovery rate (BY) was applied for *p* values, altogether 22 SNPs in *NR1D2* (*Rev-erbβ*) remained significantly associated with continuous sMEQ score and single-item chronotype, whereas all the other associations attenuated (Table 2). These significant SNPs represented 1 independent association signal (lead SNP: rs4131403).

**Table 2.** Significant clock gene associations of chronotype (BETA and OR are for A1).

						Chronotype Measure																
						Continuous sMEQ Score (*n* = 7436)					Binary sMEQ Score (*n* = 7436)						Single-Item Chronotype (Binary) (*n* = 8433)					
						(5 = Extreme Evening Type, 27 = Extreme Morning Type)					(Evening Type as Cases)						(Evening Type as Cases)					
SNP	Position	Gene	CHR	A1	MAF (A1)	BETA	SE	*P*_raw_	*P*_BH_	*P*_BY_	OR	95%CI		*P*_raw_	*P*_BH_	*P*_BY_	OR	95%CI		*P*_raw_	*P*_BH_	*P*_BY_
rs11717862	24011372	***NR1D2***	3	G	0.31	−0.22	0.07	0.002	0.009	0.040	1.10	1.02	1.18	0.019	0.059	0.281	1.11	1.04	1.19	0.002	0.006	0.027
rs13095392	24008040	***NR1D2***	3	A	0.31	−0.22	0.07	0.002	0.009	0.040	1.10	1.02	1.18	0.019	0.059	0.281	1.11	1.04	1.19	0.002	0.006	0.027
**rs4131403**	**23993437**	***NR1D2***	**3**	**A**	**0.31**	**−0.22**	**0.07**	**0.002**	**0.009**	**0.040**	**1.10**	**1.02**	**1.19**	**0.016**	**0.059**	**0.281**	**1.12**	**1.05**	**1.20**	**0.001**	**0.006**	**0.027**
rs4321479	24001078	***NR1D2***	3	C	0.30	−0.22	0.07	0.003	0.009	0.040	1.10	1.02	1.19	0.018	0.059	0.281	1.12	1.04	1.19	0.001	0.006	0.027
rs4589882	24000496	***NR1D2***	3	A	0.30	−0.22	0.07	0.003	0.009	0.040	1.10	1.02	1.19	0.018	0.059	0.281	1.12	1.04	1.19	0.001	0.006	0.027
rs4619734	24000297	***NR1D2***	3	A	0.30	−0.22	0.07	0.003	0.009	0.040	1.10	1.02	1.19	0.018	0.059	0.281	1.12	1.04	1.19	0.001	0.006	0.027
rs4858095	23996371	***NR1D2***	3	T	0.31	−0.22	0.07	0.002	0.009	0.040	1.10	1.02	1.19	0.016	0.059	0.281	1.12	1.05	1.20	0.001	0.006	0.027
rs4858098	24009919	***NR1D2***	3	G	0.31	−0.22	0.07	0.002	0.009	0.040	1.10	1.02	1.18	0.019	0.059	0.281	1.11	1.04	1.19	0.002	0.006	0.027
rs4858567	24010140	***NR1D2***	3	C	0.31	−0.22	0.07	0.002	0.009	0.040	1.10	1.02	1.18	0.019	0.059	0.281	1.11	1.04	1.19	0.002	0.006	0.027
rs5023610	24013188	***NR1D2***	3	G	0.31	−0.22	0.07	0.002	0.009	0.040	1.10	1.02	1.18	0.019	0.059	0.281	1.11	1.04	1.19	0.002	0.006	0.027
rs5847265	24013196	***NR1D2***	3	C	0.31	−0.22	0.07	0.002	0.009	0.040	1.10	1.01	1.18	0.019	0.059	0.281	1.11	1.04	1.19	0.002	0.006	0.027
rs5847266	24013250	***NR1D2***	3	G	0.31	−0.22	0.07	0.002	0.009	0.040	1.10	1.02	1.18	0.019	0.059	0.281	1.11	1.04	1.19	0.002	0.006	0.027
rs6550823	24013356	***NR1D2***	3	A	0.31	−0.22	0.07	0.002	0.009	0.040	1.10	1.02	1.18	0.019	0.059	0.281	1.11	1.04	1.19	0.002	0.006	0.027
rs6779476	24002218	***NR1D2***	3	A	0.31	−0.22	0.07	0.002	0.009	0.040	1.10	1.02	1.19	0.015	0.059	0.281	1.12	1.05	1.20	0.001	0.006	0.027
rs6790557	24005759	***NR1D2***	3	A	0.31	−0.22	0.07	0.002	0.009	0.040	1.10	1.02	1.18	0.019	0.059	0.281	1.11	1.04	1.19	0.002	0.006	0.027
rs6794922	23997978	***NR1D2***	3	G	0.31	−0.22	0.07	0.002	0.009	0.040	1.10	1.02	1.19	0.016	0.059	0.281	1.12	1.05	1.20	0.001	0.006	0.027
rs7371944	24017454	***NR1D2***	3	A	0.31	−0.22	0.07	0.002	0.009	0.040	1.10	1.02	1.18	0.019	0.059	0.281	1.11	1.04	1.19	0.002	0.006	0.027
rs7431301	24011396	***NR1D2***	3	A	0.31	−0.22	0.07	0.002	0.009	0.040	1.10	1.02	1.18	0.019	0.059	0.281	1.11	1.04	1.19	0.002	0.006	0.027
rs9882735	24014059	***NR1D2***	3	C	0.31	−0.22	0.07	0.002	0.009	0.040	1.10	1.02	1.18	0.019	0.059	0.281	1.11	1.04	1.19	0.002	0.006	0.027
rs55792500	24018503	***NR1D2***	3	G	0.31	−0.21	0.07	0.004	0.011	0.054	1.10	1.02	1.18	0.018	0.059	0.281	1.11	1.04	1.19	0.002	0.006	0.029
rs4858569	24014314	***NR1D2***	3	G	0.35	−0.21	0.07	0.003	0.009	0.042	1.10	1.02	1.18	0.016	0.059	0.281	1.09	1.02	1.16	0.010	0.029	0.139
rs7619870	23994977	***NR1D2***	3	A	0.44	−0.17	0.07	0.014	0.041	0.196	1.09	1.01	1.17	0.020	0.059	0.282	1.07	1.01	1.14	0.033	0.081	0.382

Lead SNP in bold. Adjusted for age, sex, five principal components and genotyping batch. CHR = chromosome; CI = confidence interval; MAF = minor allele frequency; OR = odds ratio; P_BH_ = Benjamini and Hochberg adjusted *p* value; P_BY_ = Benjamini and Yekutieli adjusted *p* value; sMEQ = shortened Morningness-Eveningness Questionnaire; SNP = single nucleotide polymorphism.

### GWAS of Chronotype

Our GWAS of chronotype did not yield genome-wide significant associations (*p* < 5 × 10−^8^; Suppl. Figs. 1–3). However, there were few suggestive (*p* < 1 × 10−^5^) associations found, of which 1 intergenic SNP (rs79036472) emerged in continuous and binary sMEQ scores (Suppl. Table S6; Suppl. Figs. S1–3). Furthermore, these suggestive SNPs were not among the top 10,000 genome-wide associated SNPs reported in the GWAS of Jones et al. (2019). In addition, we sought to replicate our suggestive findings in the UK Biobank data only, which was available for download at http://www.kp4cd.org/dataset_downloads/sleep. This summary data included 5309 additional genome-wide significant SNPs, which were not among the reported top 10,000 SNPs of the meta-analysis. No evidence of replication of our suggestive findings was found among those SNPs either.

### Replication of the Previous GWAS of Chronotype

We attempted to replicate the top 10,000 genome-wide associated SNPs from the previous GWAS meta-analysis of Jones et al. (2019), with *p* values <0.05 considered significant. Overall, our data included 7741 of the 10,000 SNPs, and the results did not reach statistical significance (*p* > 0.05) in any of the chronotype assessments, but the directions of the effects of the variants were mostly in the same direction (Suppl. Table S4; Suppl. Fig. S4). For the continuous sMEQ score, 80.1% (6202 of 7741) of the variants had the same direction of effect as the meta-analysis (binomial test *p* < 2.2 × 10^−16^); for the binary sMEQ score, 82.8% (6409 of 7741) of the variants had the same direction (binomial test *p* < 2.2 × 10−^16^); and for the single-item chronotype, 87.4% (6766 of 7741) of the variants had the same direction of effect as the meta-analysis (binomial test *p* < 2.2 × 10^−16^).

### GRS for Chronotype

We developed a GRS for chronotype based on 313 lead SNPs from the recent GWAS meta-analysis (Jones et al., 2019; Suppl. Table S5). Higher GRS was associated with evening type in both chronotype assessments: for the continuous sMEQ score, beta −0.49 (s.e. 0.05), *p* = 1.4 × 10^−24^; for the binary sMEQ score, odds ratio (OR) 1.24 (95% confidence interval [CI] 1.18-1.30), *p* = 1.7 × 10^−16^; for the single-item chronotype, OR 1.30 (95% CI 1.24-1.37), *p* = 4.3 × 10^−27^ (Table 3). The GRS association was strongest for the single-item chronotype, with a slightly higher proportion of variance explained (for the continuous sMEQ score: *R*^2^ = 0.01387; for the binary sMEQ score: Nagelkerke’s pseudo *R*^2^ = 0.01312; for the single-item chronotype: Nagelkerke’s pseudo *R*^2^ = 0.01893). However, no associations were found when we studied the associations between the individual SNPs of the GRS and chronotype, but directions of effects of the variants were mostly in the same direction (Suppl. Table S5; Suppl. Fig. S5). For the continuous sMEQ score, 70.9% (222 of 313) of the variants had the same direction of effect as the meta-analysis (binomial test *p* = 4.4 × 10^−14^). For the binary sMEQ score, 60.7% (190 of 313) of the variants had the same direction of effect (binomial test *p* = 9.1 × 10^−05^), and for the single-item chronotype, 72.2% (226 of 313) of the variants had the same direction of effect as the meta-analysis (binomial test *p* = 1.1 × 10^−15^).

**Table 3.** Linear or logistic regression analysis of genetic risk score (GRS) association with chronotype based on 313 chronotype-associated SNPs (Jones et al., 2019).

	GRS		
Chronotype measure	*B/*OR	SE/95% CI	*p*
Continuous sMEQ score	−0.49	0.05	1.4 × 10^−24^
Binary sMEQ score	1.24	1.18, 1.31	1.7 × 10^−16^
Single-item chronotype (binary)	1.30	1.24, 1.37	4.3 × 10^−27^

Adjusted for age, sex, 5 principal components, and genotyping batch. Decreasing beta and increasing odds ratios refer to evening type. CI = confidence interval; OR = odds ratio; sMEQ = shortened Morningness-Eveningness questionnaire.

## Discussion

To the best of our knowledge, this is the first larger-scale study on the genetic associations of chronotype with a validated chronotype questionnaire. We found a novel genetic association of chronotype with 1 clock gene *NR1D2* (*Rev-erbβ*). However, the GWAS analysis of chronotype did not yield any genome-wide significant associations; neither could we replicate any of the individual SNP associations from the previous GWAS study of chronotype (Jones et al., 2019). Furthermore, higher scores in GRS were associated with evening chronotype in our study population.

Although we could not replicate the previously reported associations between chronotype and SNPs within clock genes in our study population (e.g., Carpen et al., 2006; Etain et al., 2014; Song et al., 2016; Jones et al., 2019), we found an association between chronotype (as based on the continuous sMEQ score as well as on the single-item chronotype) and the *NR1D2* gene. Earlier, NR1D2 has been associated with a difference in timing of expression between extreme morning types and extreme evening types (Ferrante et al., 2015). The circadian clock machinery is composed of a core transcription-translation feedback loop and additional interlocking loops of transcriptional activators and repressors (Liu et al., 2008). The core feedback loop is composed of transcriptional activators CLOCK and ARNTL (BMAL1), which form a heterodimer that activates the transcription of *PER 1-2* and *CRY 1-2* genes, whose protein products form a repressor complex that inhibits their own transcription (Takahashi, 2017). The *NR1D2* gene encodes a repressor in an additional feedback loop that controls *ARNTL* transcription with retinoic acid orphan receptors (RORs) as opposing activators, where NR1D2 and NR1D1 play a more prominent role than the RORs in the basic clock mechanism to control rhythmic transcription of clock output genes (Liu et al., 2008). Furthermore, NR1D2 has a role in carbohydrate and lipid metabolism. In the liver, NR1D2 regulates hepatic lipid metabolism by repressing the expression of apolipoprotein C-III (Wang et al., 2007). In the skeletal muscle, NR1D2 controls the lipid and energy homeostasis by repressing several genes (e.g., *CD36*, *FABP3*, *UCP3*, *SCD1*, and *MSTN*) involved in lipid metabolism, body fat accumulation, and muscle hypertrophy and hyperplasia and by inducing the expression of interleukin-6 that regulates energy expenditure and inflammation (Ramakrishnan et al., 2005). Mouse studies have demonstrated that administration of synthetic NR1D2 ligands altered the expression of the circadian genes in the hypothalamus and metabolic genes in the liver, skeletal muscle, and adipose tissue, resulting in increased energy expenditure (Solt et al., 2012). Furthermore, mice with diet-induced obesity reduced fat mass and improved dyslipidemia and hyperglycemia after treatment of NR1D2 agonist (Solt et al., 2012).

These findings are supported by strong evidence from observational studies on the associations between evening chronotype and higher risks for metabolic diseases (Merikanto et al., 2013; Yu et al., 2015; Knutson and von Schantz, 2018). Furthermore, some studies have found associations between a higher body mass index or obesity and chronotype (Celis-Morales et al., 2017; de Punder et al., 2019; Sun et al., 2020), but these associations have not been confirmed among our study population (Maukonen et al., 2019). Future studies should examine in more detail the metabolic aspect of the association between *NR1D2* gene and chronotype.

All of the specific SNPs within *NR1D2* were intronic variants, and their function is unclear. Future experiments should further characterize these SNPs to inform us about their function. Furthermore, the minor allele frequencies of these SNPs were slightly lower in the Finnish population as compared with non-Finnish Europeans and the overall minor allele frequencies for all ethnic groups (https://gnomad.broadinstitute.org).

We did not find any genome-wide significant associations in our GWAS of chronotype. This could be due to our rather small sample size in terms of GWAS studies, with limited power to detect genome-wide associations. However, we found some suggestive findings, of which 1 intergenic variant (rs79036472) emerged in the analyses using continuous as well as binary sMEQ scores. Although these suggestive associations have not been found in the GWAS of Jones et al. (2019) and we were unable to replicate any of the findings from the previous GWAS, the directions of the effects of the variants were mostly in the same direction, showing a good consistency between our results and the GWAS of Jones et al. (2019).

The GRS was associated with chronotype in our study population and accounted for ~1.3% (binary sMEQ), ~1.4% (continuous sMEQ), and ~1.9% (single-item chronotype) of chronotype variation. The heritability estimates from twin studies show a range from 37% to 54% (Hur et al., 1998; Koskenvuo et al., 2007; Barclay et al., 2010; Watson et al., 2013), and in Finland, the estimate for additive genetic effects was 12% (Koskenvuo et al., 2007). The slightly stronger association found with single-item chronotype is likely because the 313 SNPs of the GRS were taken from data in which chronotype assessment was based on the single-item chronotype (Jones et al., 2019). Furthermore, because these SNPs and their effects were taken from data sets independent of ours, these results also increase the validity of our findings. The association between GRS based on external weights from independent data sets and chronotype was also found in a recent Spanish study (*n* = 1693) with overweight and obese participants with 15 SNPs that have previously been associated with chronotype (Vera et al., 2018). Together, these findings support the idea that the GRS based on GWAS studies may be a useful tool for capturing the genetic component of chronotype in different populations.

Furthermore, although we assessed chronotype with a validated and more detailed questionnaire in addition to the simple self-evaluation question on chronotype, the chronotype assessment was still based on self-report and thus is subject to possible reporting biases.

In conclusion, our findings indicated novel genetic associations of chronotype with the *NR1D2 (Rev-erbβ*) clock gene, which has a previously reported role in energy and lipid metabolism. Furthermore, GRS based on GWAS studies of chronotype may be a useful tool in capturing the genetic aspect of chronotype in different populations. However, more large-scale GWASs of chronotype are warranted in the future, with validated questionnaire-based chronotype assessments.

## Supplemental Material

Supplemental_Figure1_R1_manhattan – Supplemental material for Genetic Associations of Chronotype in the Finnish General Population Click here for additional data file. Supplemental material, Supplemental_Figure1_R1_manhattan for Genetic Associations of Chronotype in the Finnish General Population by Mirkka Maukonen, Aki S. Havulinna, Satu Männistö, Noora Kanerva, Veikko Salomaa and Timo Partonen in Journal of Biological Rhythms

Supplemental_Figure2_R1_manhattan – Supplemental material for Genetic Associations of Chronotype in the Finnish General Population Click here for additional data file. Supplemental material, Supplemental_Figure2_R1_manhattan for Genetic Associations of Chronotype in the Finnish General Population by Mirkka Maukonen, Aki S. Havulinna, Satu Männistö, Noora Kanerva, Veikko Salomaa and Timo Partonen in Journal of Biological Rhythms

Supplemental_Figure3_R1_manhattan – Supplemental material for Genetic Associations of Chronotype in the Finnish General Population Click here for additional data file. Supplemental material, Supplemental_Figure3_R1_manhattan for Genetic Associations of Chronotype in the Finnish General Population by Mirkka Maukonen, Aki S. Havulinna, Satu Männistö, Noora Kanerva, Veikko Salomaa and Timo Partonen in Journal of Biological Rhythms

Supplemental_Figure4_R1_scatterplot – Supplemental material for Genetic Associations of Chronotype in the Finnish General Population Click here for additional data file. Supplemental material, Supplemental_Figure4_R1_scatterplot for Genetic Associations of Chronotype in the Finnish General Population by Mirkka Maukonen, Aki S. Havulinna, Satu Männistö, Noora Kanerva, Veikko Salomaa and Timo Partonen in Journal of Biological Rhythms

Supplemental_Figure5_R1_scatterplot – Supplemental material for Genetic Associations of Chronotype in the Finnish General Population Click here for additional data file. Supplemental material, Supplemental_Figure5_R1_scatterplot for Genetic Associations of Chronotype in the Finnish General Population by Mirkka Maukonen, Aki S. Havulinna, Satu Männistö, Noora Kanerva, Veikko Salomaa and Timo Partonen in Journal of Biological Rhythms

Supplemental_Table2_R2 – Supplemental material for Genetic Associations of Chronotype in the Finnish General Population Click here for additional data file. Supplemental material, Supplemental_Table2_R2 for Genetic Associations of Chronotype in the Finnish General Population by Mirkka Maukonen, Aki S. Havulinna, Satu Männistö, Noora Kanerva, Veikko Salomaa and Timo Partonen in Journal of Biological Rhythms

Supplemental_Table3_R2 – Supplemental material for Genetic Associations of Chronotype in the Finnish General Population Click here for additional data file. Supplemental material, Supplemental_Table3_R2 for Genetic Associations of Chronotype in the Finnish General Population by Mirkka Maukonen, Aki S. Havulinna, Satu Männistö, Noora Kanerva, Veikko Salomaa and Timo Partonen in Journal of Biological Rhythms

Supplemental_Table4_R2 – Supplemental material for Genetic Associations of Chronotype in the Finnish General Population Click here for additional data file. Supplemental material, Supplemental_Table4_R2 for Genetic Associations of Chronotype in the Finnish General Population by Mirkka Maukonen, Aki S. Havulinna, Satu Männistö, Noora Kanerva, Veikko Salomaa and Timo Partonen in Journal of Biological Rhythms

Supplemental_Table5_R2 – Supplemental material for Genetic Associations of Chronotype in the Finnish General Population Click here for additional data file. Supplemental material, Supplemental_Table5_R2 for Genetic Associations of Chronotype in the Finnish General Population by Mirkka Maukonen, Aki S. Havulinna, Satu Männistö, Noora Kanerva, Veikko Salomaa and Timo Partonen in Journal of Biological Rhythms

Supplemental_Table6_R2 – Supplemental material for Genetic Associations of Chronotype in the Finnish General Population Click here for additional data file. Supplemental material, Supplemental_Table6_R2 for Genetic Associations of Chronotype in the Finnish General Population by Mirkka Maukonen, Aki S. Havulinna, Satu Männistö, Noora Kanerva, Veikko Salomaa and Timo Partonen in Journal of Biological Rhythms

Supplemental_Table_1_R2 – Supplemental material for Genetic Associations of Chronotype in the Finnish General Population Click here for additional data file. Supplemental material, Supplemental_Table_1_R2 for Genetic Associations of Chronotype in the Finnish General Population by Mirkka Maukonen, Aki S. Havulinna, Satu Männistö, Noora Kanerva, Veikko Salomaa and Timo Partonen in Journal of Biological Rhythms

## References

[bibr1-0748730420935328] Ashbrook LH Krystal AD Fu YH Ptacek LJ (2020). Genetics of the human circadian clock and sleep homeostat. Neuropsychopharmacology 45:45-54. 31400754 10.1038/s41386-019-0476-7 PMC6879540

[bibr2-0748730420935328] Barclay NL Eley TC Buysse DJ Archer SN Gregory AM (2010) Diurnal preference and sleep quality: same genes? A study of young adult twins. Chronobiol Int 27:278-296. 20370470 10.3109/07420521003663801

[bibr3-0748730420935328] Barclay NL Watson NF Buchwald D Goldberg J (2014) Moderation of genetic and environmental influences on diurnal preference by age in adult twins. Chronobiol Int 31:222-231. 24128196 10.3109/07420528.2013.842924

[bibr4-0748730420935328] Borodulin K Vartiainen E Peltonen M Jousilahti P Juolevi A Laatikainen T Männistö S Salomaa V Sundvall J Puska P (2015) Forty-year trends in cardiovascular risk factors in Finland. Eur J Public Health 25:539-546. 25422363 10.1093/eurpub/cku174

[bibr5-0748730420935328] Broms U Pitkäniemi J Backmand H Heikkilä K Koskenvuo M Peltonen M Sarna S Vartiainen E Kaprio J Partonen T (2014) Long-term consistency of diurnal-type preferences among men. Chronobiol Int 31:182-188. 24131152 10.3109/07420528.2013.836534

[bibr6-0748730420935328] Carpen JD Archer SN Skene DJ Smits M von Schantz M (2005) A single-nucleotide polymorphism in the 5′-untranslated region of the hPER2 gene is associated with diurnal preference. J Sleep Res 14:293-297. 16120104 10.1111/j.1365-2869.2005.00471.x

[bibr7-0748730420935328] Carpen JD von Schantz M Smits M Skene DJ Archer SN (2006) A silent polymorphism in the PER1 gene associates with extreme diurnal preference in humans. J Hum Genet 51:1122-1125. 17051316 10.1007/s10038-006-0060-y

[bibr8-0748730420935328] Celis-Morales C Lyall DM Guo Y Steell L Llanas D Ward J Mackay DF Biello SM Bailey ME Pell JP, et al (2017) Sleep characteristics modify the association of genetic predisposition with obesity and anthropometric measurements in 119,679 UK Biobank participants. Am J Clin Nutr 105:980-990. 28251931 10.3945/ajcn.116.147231

[bibr9-0748730420935328] Chang CC Chow CC Tellier LC Vattikuti S Purcell SM Lee JJ (2015) Second-generation PLINK: rising to the challenge of larger and richer datasets. Gigascience 4:7-8. 25722852 10.1186/s13742-015-0047-8 PMC4342193

[bibr10-0748730420935328] de Punder K Heim C Entringer S (2019) Association between chronotype and body mass index: the role of C-reactive protein and the cortisol response to stress. Psychoneuroendocrinology 109:104388. 31398588 10.1016/j.psyneuen.2019.104388

[bibr11-0748730420935328] Didikoglu A Maharani A Payton A Pendleton N Canal MM (2019) Longitudinal change of sleep timing: association between chronotype and longevity in older adults. Chronobiol Int 36:1285-1300. 31328571 10.1080/07420528.2019.1641111

[bibr12-0748730420935328] Dmitrzak-Weglarz M Pawlak J Wilkosc M Miechowicz I Maciukiewicz M Ciarkowska W Zaremba D Hauser J (2016) Chronotype and sleep quality as a subphenotype in association studies of clock genes in mood disorders. Acta Neurobiol Exp (Wars) 76:32-42. 27102916 10.21307/ane-2017-003

[bibr13-0748730420935328] Etain B Jamain S Milhiet V Lajnef M Boudebesse C Dumaine A Mathieu F Gombert A Ledudal K Gard S, et al (2014) Association between circadian genes, bipolar disorders and chronotypes. Chronobiol Int 31:807-814. 24716566 10.3109/07420528.2014.906445

[bibr14-0748730420935328] Ferrante A Gellerman D Ay A Woods KP Filipowicz AM Jain K Bearden N Ingram KK (2015) Diurnal preference predicts phase differences in expression of human peripheral circadian clock genes. J Circadian Rhythms 13:4. 27103930 10.5334/jcr.ae PMC4832819

[bibr15-0748730420935328] Hätönen T Forsblom S Kieseppä T Lönnqvist J Partonen T (2008) Circadian phenotype in patients with the co-morbid alcohol use and bipolar disorders. Alcohol Alcohol 43:564-568. 18644800 10.1093/alcalc/agn057

[bibr16-0748730420935328] Hayes KR Baggs JE Hogenesch JB (2005) Circadian clocks are seeing the systems biology light. Genome Biol 6:219-219. 15892879 10.1186/gb-2005-6-5-219 PMC1175949

[bibr17-0748730420935328] Horne JA Östberg O (1976) A self-assessment questionnaire to determine morningness-eveningness in human circadian rhythms. Int J Chronobiol 4:97-110. 1027738

[bibr18-0748730420935328] Howie BN Donnelly P Marchini J (2009) A flexible and accurate genotype imputation method for the next generation of genome-wide association studies. PLoS Genet. 5:e1000529. 19543373 10.1371/journal.pgen.1000529 PMC2689936

[bibr19-0748730420935328] Hu Y Shmygelska A Tran D Eriksson N Tung JY Hinds DA (2016) GWAS of 89,283 individuals identifies genetic variants associated with self-reporting of being a morning person. Nat Commun 7:10448. 26835600 10.1038/ncomms10448 PMC4740817

[bibr20-0748730420935328] Hur YM Bouchard TJ Lykken DT (1998) Genetic and environmental influence on morningness–eveningnessfn2fn2Part of the material reported here was presented at the 27th annual meeting of the Behavior Genetics Association. Personality and Individual Differences 25:917-925.

[bibr21-0748730420935328] Jankowski KS Dmitrzak-Weglarz M (2017) ARNTL, CLOCK and PER3 polymorphisms: links with chronotype and affective dimensions. Chronobiol Int 34:1105-1113. 28708003 10.1080/07420528.2017.1343341

[bibr22-0748730420935328] Jones SE Lane JM Wood AR van Hees VT Tyrrell J Beaumont RN Jeffries AR Dashti HS Hillsdon M Ruth KS, et al (2019) Genome-wide association analyses of chronotype in 697,828 individuals provides insights into circadian rhythms. Nat Commun 10:343-347. 30696823 10.1038/s41467-018-08259-7 PMC6351539

[bibr23-0748730420935328] Jones SE Tyrrell J Wood AR Beaumont RN Ruth KS Tuke MA Yaghootkar H Hu Y Teder-Laving M Hayward C, et al (2016) Genome-wide association analyses in 128,266 individuals identifies new morningness and sleep duration loci. PLoS Genet 12:e1006125. 27494321 10.1371/journal.pgen.1006125 PMC4975467

[bibr24-0748730420935328] Kanerva N Kronholm E Partonen T Ovaskainen ML Kaartinen NE Konttinen H Broms U Männistö S (2012) Tendency toward eveningness is associated with unhealthy dietary habits. Chronobiol Int 29:920-927. 22823875 10.3109/07420528.2012.699128

[bibr25-0748730420935328] Katzenberg D Young T Finn L Lin L King DP Takahashi JS Mignot E (1998).A CLOCK polymorphism associated with human diurnal preference. Sleep 21:569-576. 9779516 10.1093/sleep/21.6.569

[bibr26-0748730420935328] Knutson KL von Schantz M (2018) Associations between chronotype, morbidity and mortality in the UK Biobank cohort. Chronobiol Int 35:1045-1053. 29642757 10.1080/07420528.2018.1454458 PMC6119081

[bibr27-0748730420935328] Koskenvuo M Hublin C Partinen M Heikkilä K Kaprio J (2007) Heritability of diurnal type: a nationwide study of 8753 adult twin pairs. J Sleep Res 16:156-162. 17542945 10.1111/j.1365-2869.2007.00580.x

[bibr28-0748730420935328] Kripke DF Klimecki WT Nievergelt CM Rex KM Murray SS Shekhtman T Tranah GJ Loving RT Lee HJ Rhee MK, et al (2014) Circadian polymorphisms in night owls, in bipolars, and in non-24-hour sleep cycles. Psychiatry Investig 11:345-362. 10.4306/pi.2014.11.4.345 PMC4225198 25395965

[bibr29-0748730420935328] Kurien P Hsu PK Leon J Wu D McMahon T Shi G Xu Y Lipzen A Pennacchio LA Jones CR, et al (2019) TIMELESS mutation alters phase responsiveness and causes advanced sleep phase. Proc Natl Acad Sci USA 116:12045-12053. 31138685 10.1073/pnas.1819110116 PMC6575169

[bibr30-0748730420935328] Lane JM Vlasac I Anderson SG Kyle SD Dixon WG Bechtold DA Gill S Little MA Luik A Loudon A, et al (2016) Genome-wide association analysis identifies novel loci for chronotype in 100,420 individuals from the UK Biobank. Nat Commun 7:10889. 26955885 10.1038/ncomms10889 PMC4786869

[bibr31-0748730420935328] Lee HJ Kim L Kang SG Yoon HK Choi JE Park YM Kim SJ Kripke DF (2011) PER2 variation is associated with diurnal preference in a Korean young population. Behav Genet 41:273-277. 20931356 10.1007/s10519-010-9396-3 PMC3044841

[bibr32-0748730420935328] Levandovski R Dantas G Fernandes LC Caumo W Torres I Roenneberg T Hidalgo MP Allebrandt KV (2011) Depression scores associate with chronotype and social jetlag in a rural population. Chronobiol Int 28:771-778. 21895489 10.3109/07420528.2011.602445

[bibr33-0748730420935328] Liu AC Tran HG Zhang EE Priest AA Welsh DK Kay SA (2008) Redundant function of REV-ERBalpha and beta and non-essential role for Bmal1 cycling in transcriptional regulation of intracellular circadian rhythms. PLoS Genet 4:e1000023. 18454201 10.1371/journal.pgen.1000023 PMC2265523

[bibr34-0748730420935328] Locke AE Steinberg KM Chiang CWK Service SK Havulinna AS Stell L Pirinen M Abel HJ Chiang CC Fulton RS, et al (2019). Exome sequencing of Finnish isolates enhances rare-variant association power. Nature 572:323-328. 31367044 10.1038/s41586-019-1457-z PMC6697530

[bibr35-0748730420935328] Loh P-R Danecek P Palamara PF Fuchsberger C Reshef YA Finucane HK Schoenherr S Forer L McCarthy S Abecasis GR, et al (2016) Reference-based phasing using the Haplotype Reference Consortium panel. Nat Genet 48:1443-1448. 27694958 10.1038/ng.3679 PMC5096458

[bibr36-0748730420935328] Matsuo M Shiino Y Yamada N Ozeki Y Okawa M (2007) A novel SNP in hPer2 associates with diurnal preference in a healthy population. Sleep Biol Rhythms 5:141-145.

[bibr37-0748730420935328] Maukonen M Kanerva N Partonen T Kronholm E Tapanainen H Kontto J Männistö S (2017) Chronotype differences in timing of energy and macronutrient intakes: a population-based study in adults. Obesity (Silver Spring) 25:608-615. 28229553 10.1002/oby.21747

[bibr38-0748730420935328] Maukonen M Kanerva N Partonen T Männistö S (2019) Chronotype and energy intake timing in relation to changes in anthropometrics: a 7-year follow-up study in adults. Chronobiol Int 36:27-41. 30212231 10.1080/07420528.2018.1515772

[bibr39-0748730420935328] Merikanto I Kronholm E Peltonen M Laatikainen T Vartiainen E Partonen T (2015) Circadian preference links to depression in general adult population. J Affect Disord 188:143-148. 26363264 10.1016/j.jad.2015.08.061

[bibr40-0748730420935328] Merikanto I Lahti T Puolijoki H Vanhala M Peltonen M Laatikainen T Vartiainen E Salomaa V Kronholm E Partonen T (2013) Associations of chronotype and sleep with cardiovascular diseases and type 2 diabetes. Chronobiol Int 30:470-477. 23281716 10.3109/07420528.2012.741171

[bibr41-0748730420935328] Mishima K Tozawa T Satoh K Saitoh H Mishima Y (2005) The 3111T/C polymorphism of hClock is associated with evening preference and delayed sleep timing in a Japanese population sample. Am J Med Genet B Neuropsychiatr Genet 2005;133B:101-104. 10.1002/ajmg.b.30110 15578592

[bibr42-0748730420935328] Parsons MJ Lester KJ Barclay NL Archer SN Nolan PM Eley TC Gregory AM (2014) Polymorphisms in the circadian expressed genes PER3 and ARNTL2 are associated with diurnal preference and GNbeta3 with sleep measures. J Sleep Res 23:595-604. 24635757 10.1111/jsr.12144 PMC4320759

[bibr43-0748730420935328] Patke A Young MW Axelrod S (2019) Molecular mechanisms and physiological importance of circadian rhythms. Nat Rev Mol Cell Biol 21:67-84. 31768006 10.1038/s41580-019-0179-2

[bibr44-0748730420935328] Patterson F Malone SK Grandner MA Lozano A Perkett M Hanlon A (2018) Interactive effects of sleep duration and morning/evening preference on cardiovascular risk factors. Eur J Public Health 28:155-161. 28371850 10.1093/eurpub/ckx029 PMC5881732

[bibr45-0748730420935328] Patterson F Malone SK Lozano A Grandner MA Hanlon AL (2016) Smoking, screen-based sedentary behavior, and diet associated with habitual sleep duration and chronotype: data from the UK Biobank. Ann Behav Med 50:715-726. 27056396 10.1007/s12160-016-9797-5 PMC5079686

[bibr46-0748730420935328] Ramakrishnan SN Lau P Burke LJ Muscat GE (2005) Rev-erbbeta regulates the expression of genes involved in lipid absorption in skeletal muscle cells: evidence for cross-talk between orphan nuclear receptors and myokines. J Biol Chem 280:8651-8659. 15623503 10.1074/jbc.M413949200

[bibr47-0748730420935328] R Core Team (2018) R: A Language and Environment for Statistical Computing. Vienna (Austria): R Foundation for Statistical Computing https://www.R-project.org/.

[bibr48-0748730420935328] Reinke H Asher G (2019) Crosstalk between metabolism and circadian clocks. Nat Rev Mol Cell Biol 20:227-241. 30635659 10.1038/s41580-018-0096-9

[bibr49-0748730420935328] Ribas-Latre A Eckel-Mahan K (2016) Interdependence of nutrient metabolism and the circadian clock system: importance for metabolic health. Mol Metab 5:133-152. 26977390 10.1016/j.molmet.2015.12.006 PMC4770266

[bibr50-0748730420935328] Roenneberg T Kuehnle T Juda M Kantermann T Allebrandt K Gordijn M Merrow M (2007) Epidemiology of the human circadian clock. Sleep Med Rev 2007;11:429-438. 10.1016/j.smrv.2007.07.005 17936039

[bibr51-0748730420935328] Sato F Kohsaka A Bhawal UK Muragaki Y (2018) Potential roles of Dec and Bmal1 genes in interconnecting circadian clock and energy metabolism. Int J Mol Sci 19:781. 10.3390/ijms19030781 PMC5877642 29518061

[bibr52-0748730420935328] Sato-Mito N Sasaki S Murakami K Okubo H Takahashi Y Shibata S Yamada K Sato K; Freshmen in Dietetic Courses Study II Group (2011) The midpoint of sleep is associated with dietary intake and dietary behavior among young Japanese women. Sleep Med 12:289-294. 21296614 10.1016/j.sleep.2010.09.012

[bibr53-0748730420935328] Solt LA Wang Y Banerjee S Hughes T Kojetin DJ Lundasen T Shin Y Liu J Cameron MD Noel R, et al (2012) Regulation of circadian behaviour and metabolism by synthetic REV-ERB agonists. Nature 485:62-68. 22460951 10.1038/nature11030 PMC3343186

[bibr54-0748730420935328] Song HM Cho CH Lee HJ Moon JH Kang SG Yoon HK Park YM Kim L (2016) Association of CLOCK, ARNTL, PER2, and GNB3 polymorphisms with diurnal preference in a Korean population. Chronobiol Int 33:1455-1463. 27660894 10.1080/07420528.2016.1231199

[bibr55-0748730420935328] Sun X Gustat J Bertisch SM Redline S Bazzano L (2020) The association between sleep chronotype and obesity among black and white participants of the Bogalusa Heart Study. Chronobiol Int 37:123-134. 31747792 10.1080/07420528.2019.1689398 PMC6981036

[bibr56-0748730420935328] Takahashi JS (2017). Transcriptional architecture of the mammalian circadian clock. Nat Rev Genet 18:164-179. 27990019 10.1038/nrg.2016.150 PMC5501165

[bibr57-0748730420935328] Vartiainen E Laatikainen T Peltonen M Juolevi A Männistö S Sundvall J Jousilahti P Salomaa V Valsta L Puska P (2010) Thirty-five-year trends in cardiovascular risk factors in Finland. Int J Epidemiol 39:504-518. 19959603 10.1093/ije/dyp330

[bibr58-0748730420935328] Vera B Dashti HS Gómez-Abellán P Hernández-Martínez AM (2018) Modifiable lifestyle behaviors, but not a genetic risk score, associate with metabolic syndrome in evening chronotypes. Sci Rep 8:945. 29343740 10.1038/s41598-017-18268-z PMC5772646

[bibr59-0748730420935328] von Schantz M (2017) Natural variation in human clocks. Adv Genet 99:73-96. 29050555 10.1016/bs.adgen.2017.09.003

[bibr60-0748730420935328] Wang J Li Y Zhang M Liu Z Wu C Yuan H Li YY Zhao X Lu H (2007) A zinc finger HIT domain-containing protein, ZNHIT-1, interacts with orphan nuclear hormone receptor Rev-erbbeta and removes Rev-erbbeta-induced inhibition of apoCIII transcription. FEBS J 274:5370-5381. 17892483 10.1111/j.1742-4658.2007.06062.x

[bibr61-0748730420935328] Watson NF Buchwald D Harden KP (2013) A twin study of genetic influences on diurnal preference and risk for alcohol use outcomes. J Clin Sleep Med 9:1333-1339. 24340296 10.5664/jcsm.3282 PMC3836345

[bibr62-0748730420935328] Wennman H Kronholm E Partonen T Peltonen M Vasankari T Borodulin K (2015) Evening typology and morning tiredness associates with low leisure time physical activity and high sitting. Chronobiol Int 32:1090-1100. 26317556 10.3109/07420528.2015.1063061

[bibr63-0748730420935328] Yu JH Yun CH Ahn JH Suh S Cho HJ Lee SK Yoo HJ Seo JA Kim SG Choi KM, et al (2015) Evening chronotype is associated with metabolic disorders and body composition in middle-aged adults. J Clin Endocrinol Metab 100:1494-1502. 25831477 10.1210/jc.2014-3754

