# Supplementary figures and images for: Genetic Associations of Chronotype in the Finnish General Population
Source: J Biol Rhythms. 2020 Jun 24;35(5):501–11. doi: 10.1177/0748730420935328 (PMC7534025; doi:10.1177/0748730420935328)

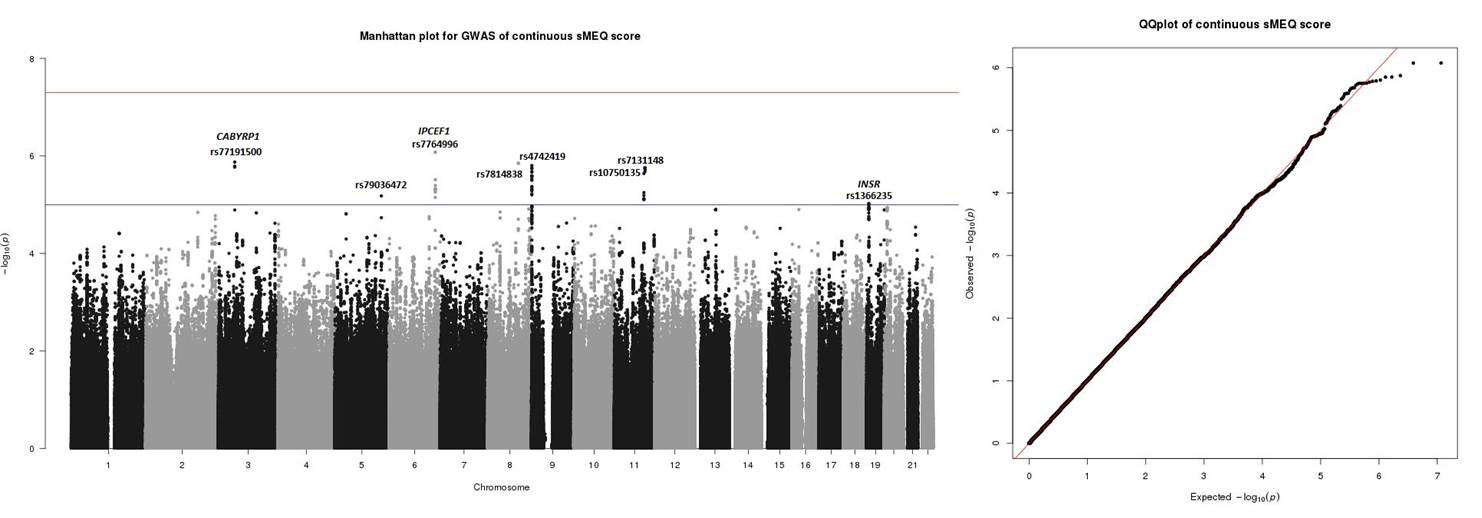

Supplement: Supplemental_Figure1_R1_manhattan – Supplemental material for Genetic Associations of Chronotype in the Finnish General Population [file Supplemental_Figure1_R1_manhattan.jpg]

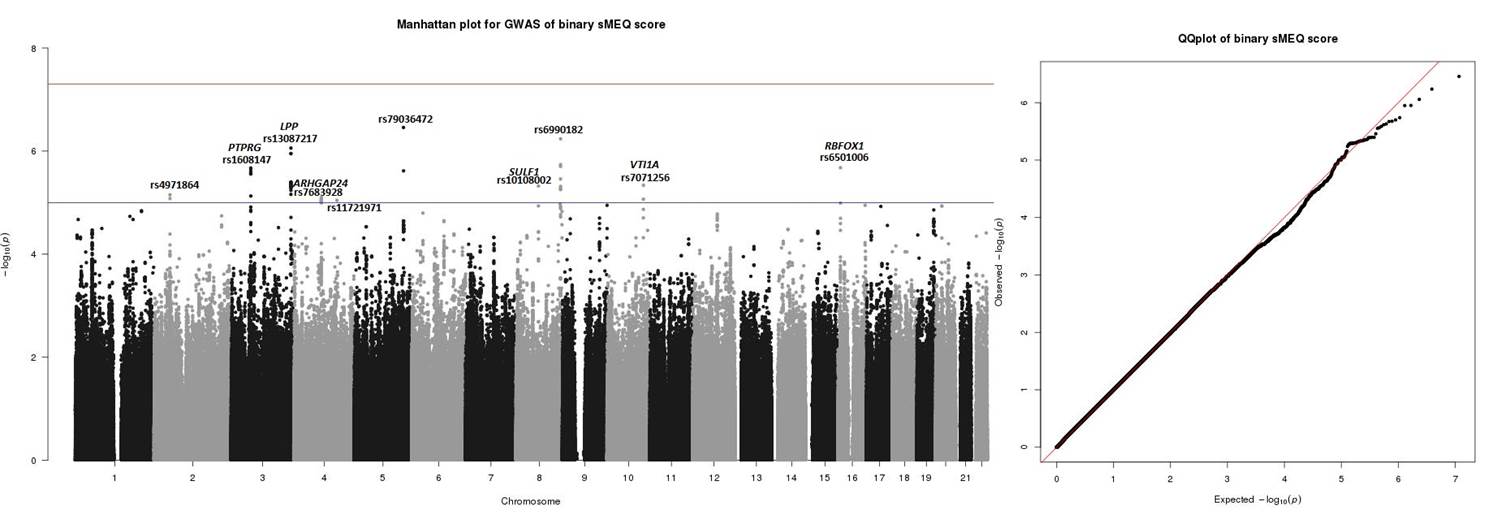

Supplement: Supplemental_Figure2_R1_manhattan – Supplemental material for Genetic Associations of Chronotype in the Finnish General Population [file Supplemental_Figure2_R1_manhattan.jpg]

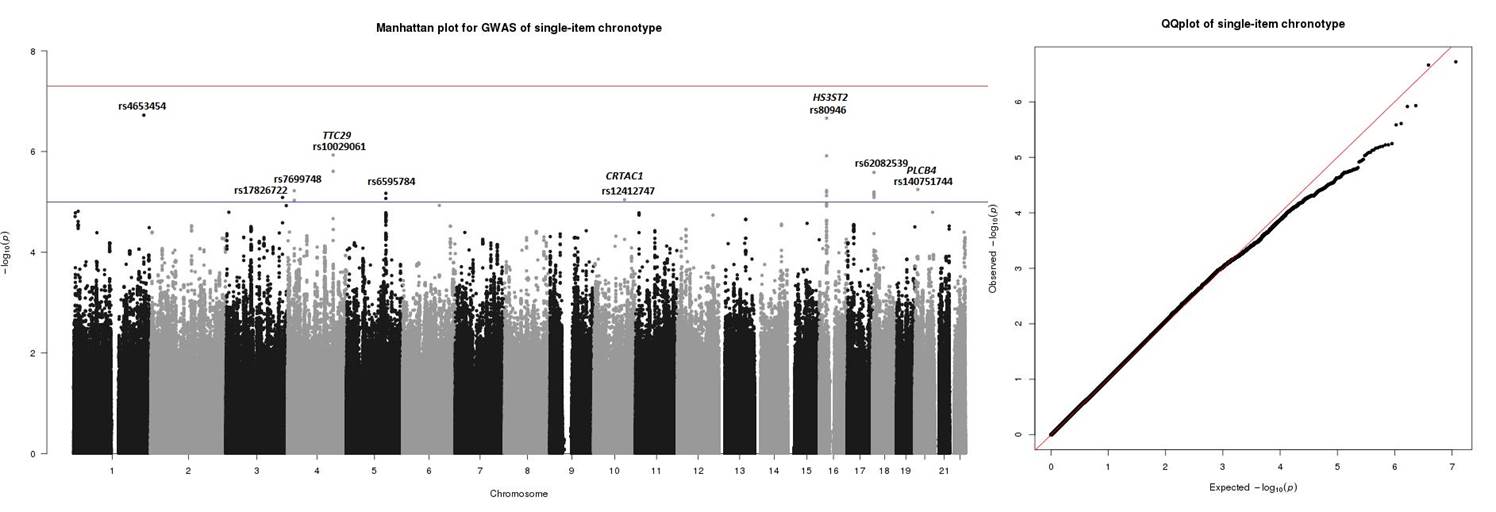

Supplement: Supplemental_Figure3_R1_manhattan – Supplemental material for Genetic Associations of Chronotype in the Finnish General Population [file Supplemental_Figure3_R1_manhattan.jpg]

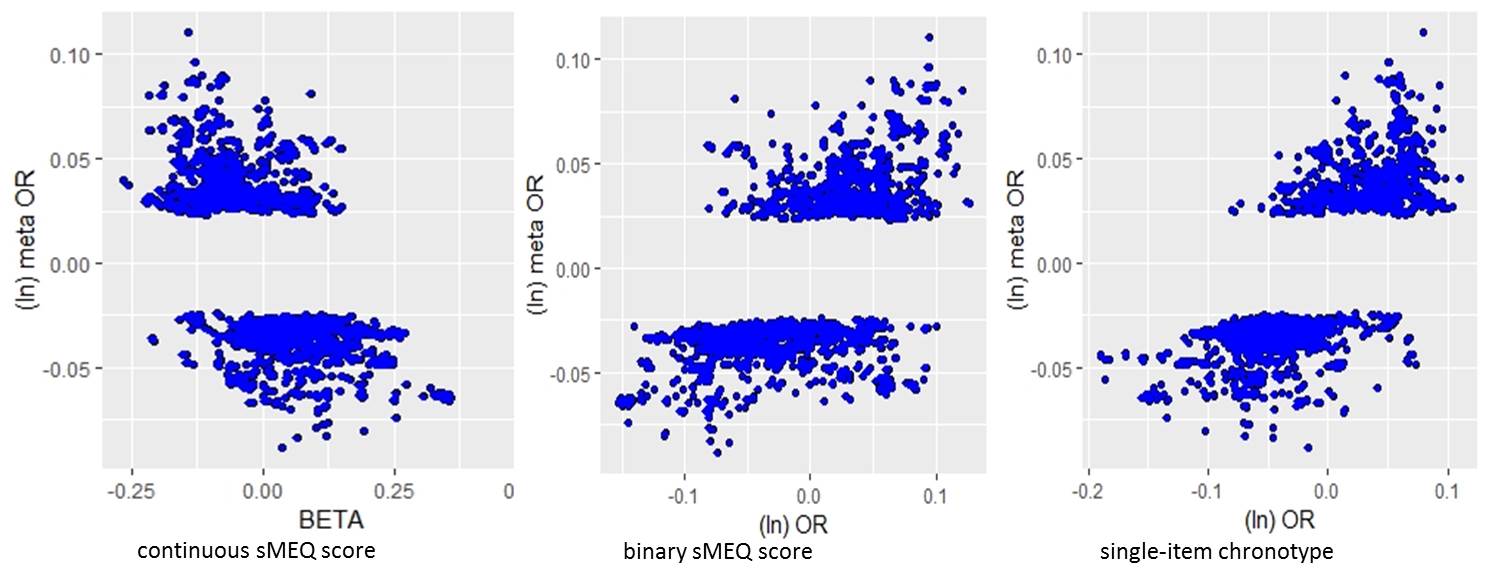

Supplement: Supplemental_Figure4_R1_scatterplot – Supplemental material for Genetic Associations of Chronotype in the Finnish General Population [file Supplemental_Figure4_R1_scatterplot.jpg]

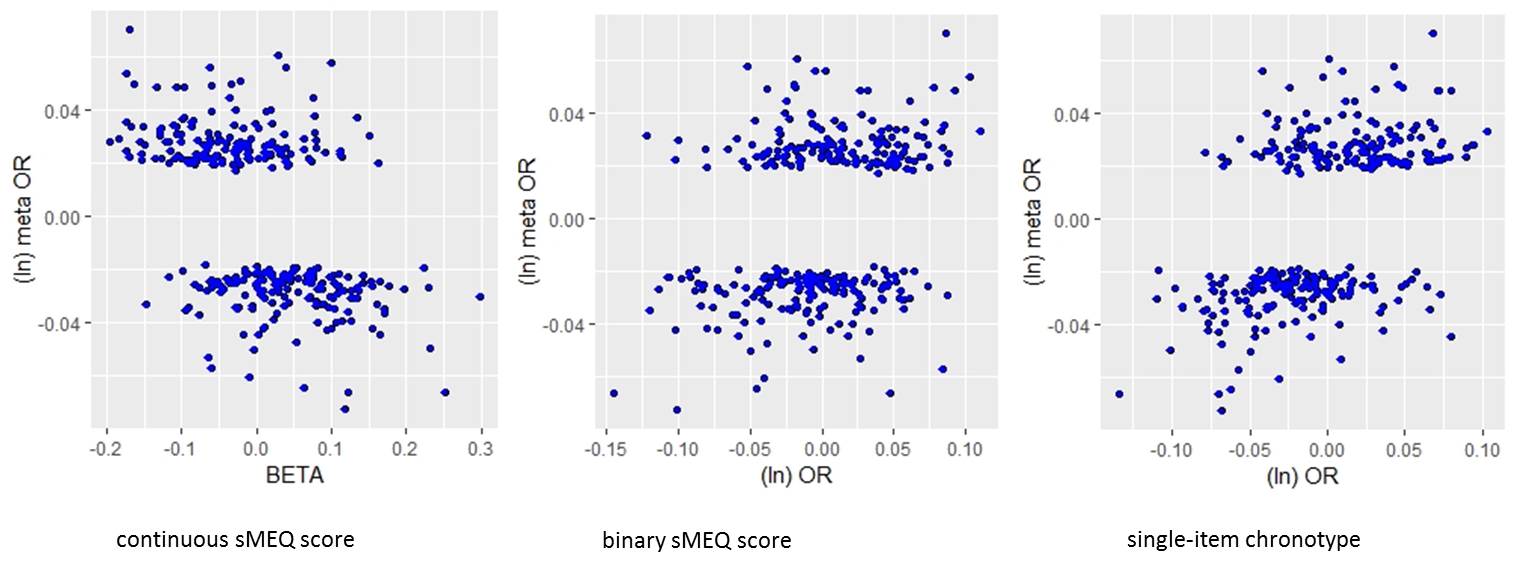

Supplement: Supplemental_Figure5_R1_scatterplot – Supplemental material for Genetic Associations of Chronotype in the Finnish General Population [file Supplemental_Figure5_R1_scatterplot.jpg]
