# Supplementary material for: Genetic Associations of Chronotype in the Finnish General Population
Source: J Biol Rhythms. 2020 Jun 24;35(5):501–11. doi: 10.1177/0748730420935328 (PMC7534025; doi:10.1177/0748730420935328)
Supplement: Supplemental_Table_1_R2 – Supplemental material for Genetic Associations of Chronotype in the Finnish General Population [file Supplemental_Table_1_R2.doc]

| Supplemental Table 1. A shortened six-item version of Horne and Östberg’s (1976) Morningness-Eveningness Questionnaire. |
| --- |
| *Item 4* |
| "Assuming adequate environmental conditions, how easy do you find getting up in the morning?" |
| 1=“not easy at all” |
| 2="not very easy" |
| 3="quite easy" |
| 4=“very easy" |
|  |
| *Item 7* |
| “During the first half-hour after woken up in the morning, how tired do you feel?” |
| 1=“very tired” |
| 2= "quite tired" |
| 3= "quite rested" |
| 4=“very rested” |
|  |
| *Item 9* |
| “You have decided to engage some physical exercise. A friend suggests that you do this one hour twice a week and the best time for your friend is between 7.0- 8.0 AM. Bearing in mind nothing else but your “feeling best” rhythm, how do you think you would perform?” |
| 1= “Would be in good form” |
| 2= "Would be in moderate form" |
| 3= "Would find it quite difficult" |
| 4= “Would find it very difficult” |
|  |
| *Item 15* |
| “You have to do two hours of hard physical work. Considering only your own “feeling best” rhythm, which of these following times would you choose?” |
| 1=“8:00-10:00” |
| 2=“11:00-13:00” |
| 3=“15:00-17:00” |
| 4=“19:00-21:00” |
|  |
| *Item 17* |
| “Suppose you can choose your own work hours. Assume that you worked a five hour day. Which five consecutive hours would you select?" |
| "1-2", "2-3", "3-4", "4-5", "5-6", "6-7", "7-8", "8-9", "9-10", "10-11", "11-12", "12-13", "13-14", "14-15", "15-16", "16-17", "17-18", "18-19", "19-20", "20-21", "21-22", "22-23", "23-24", "24-01" |
|  |
| *Item 19* |
| “There are so called morning people and evening people, which are you?” |
| 1=“definitely a morning type” |
| 2=“rather more a morning than an evening type” |
| 3= “rather more an evening than a morning type” |
| 4=“definitely an evening type” |
